# Categories of anxiety based on latent class analysis in gestational diabetes mellitus and associated factors: A questionnaire-based, cross-sectional study

**DOI:** 10.1097/MD.0000000000039168

**Published:** 2024-08-09

**Authors:** Hong Qin, Fulan Wang, Lin Wang

**Affiliations:** a Nursing Department, The First Affiliated Hospital of Chongqing Medical University, Chongqing, China; b Department of Obstetrics, The First Affiliated Hospital of Chongqing Medical University, Chongqing, China.

**Keywords:** anxiety, gestational diabetes mellitus, husband and wife relationship, influencing factors, potential category analysis

## Abstract

To investigate the classification of anxiety based on potential category analysis in gestational diabetes mellitus (GDM) patients and the associated factors. This questionnaire-based, cross-sectional study was conducted on GDM patients admitted to a Grade III-A general hospital using convenience sampling between March and November 2021. Latent class analysis was utilized for classification. Multivariate logistic regression analysis was performed to identify factors associated with anxiety. A total of 215 valid questionnaires were collected, yielding a response rate of 99%. GDM patients were classified into 4 potential categories: low anxiety (54%), high anxiety (21%), worried about the fetus (11%), and worried about delivery (14%). Multivariate logistic regression analysis showed that, compared with low anxiety, education level, family history of diabetes, blood glucose changes, delivery mode schedule, knowledge score of GDM, and marital relationship scale score were independently associated with anxiety (*P* < .05). The number of births, education level, blood glucose changes, delivery mode schedule, and marital relationship scale score were independently associated with being worried about the fetus (*P* < .05). Education level, family history of diabetes, blood glucose changes, delivery mode schedule, knowledge score of GDM, and marital relationship scale score were independently associated with being worried about delivery (*P* < .05). Anxiety in GDM patients was categorized by latent class analysis into low anxiety (54%), high anxiety (21%), worried about the fetus (11%), and worried about delivery (14%). Education level, family history of diabetes, blood glucose changes, delivery mode schedule, GDM knowledge score, and marital relationship scale score might be associated with anxiety.

## 1. Introduction

Gestational diabetes mellitus (GDM) is characterized by carbohydrate intolerance during pregnancy.^[1]^ GDM is one of the most common complications of gestation, resulting in various fetal disorders, including macrosomia, fetal malformation, fetal growth restriction, abortion, and even fetal death in utero.^[2]^ Moreover, GDM negatively affects the health of women. Women with GDM are reported to have a substantially high risk of developing type 2 diabetes after pregnancy.^[3]^ In China, the prevalence of GDM ranges from 17.5% to 18.9% and is continuously increasing.^[4]^

GDM patients often experience significant anxiety due to the increased clinical risks to both mother and child resulting from this condition.^[5]^ Studies have shown that the incidence of anxiety among GDM patients is between 43.1% and 59%, which is significantly higher than that among normal pregnant women.^[6,7]^ Pregnancy-related anxiety can lead to a range of adverse outcomes such as postpartum depression, fetal growth restriction, preterm birth, increased risk of neonatal diseases, and even suicidal ideation.^[8,9]^ This poses a serious threat to the safety of both mother and infant. According to the “Healthy China 2030” project, maintaining the safety and health of both mother and infant is a solid foundation.^[10]^ Therefore, early detection and management of prenatal anxiety in patients are of crucial importance. According to a study conducted by Kasalova, dissatisfaction in a relationship can trigger the development of anxiety disorders and may also be responsible for the modulation and maintenance of anxiety.^[11]^ However, most previous research on anxiety among GDM patients has mainly focused on individual factors, neglecting the exploration of marital relationships. A meta-analysis reported that approximately 20% of women experience anxiety disorders during pregnancy or in the postpartum period.^[12]^ Conversely, another meta-analysis showed that approximately 8.5% of postpartum women had anxiety disorders.^[13]^ The variation in the incidence of anxiety during pregnancy or the postpartum period may result from subtle differences in the detection and diagnosis of peripartum anxiety disorders. However, most previous studies relied solely on total anxiety scores to assess anxiety, overlooking its potential heterogeneity. Thus, it is essential to achieve a precise diagnosis and grading of anxiety.

Latent class analysis (LCA) is a statistical technique that categorizes individuals into categories based on their item response probabilities. This allows for the identification of latent categories and a better understanding of the distribution and proportions of different individual types. This approach can facilitate targeted and precise intervention for different categories with distinct characteristics.^[14]^ A study conducted by Beijers showed that different biomarkers classified by LCA could be used to identify various patient clusters,^[15]^ indicating that LCA can be used as a reliable method for classification.

This study aimed to investigate the classification of anxiety based on potential category analysis of GDM and associated factors. This study provides theoretical guidance and directions for the prevention and treatment of anxiety in patients with GDM.

## 2. Methods

### 2.1. Study design and participants

This questionnaire-based, cross-sectional study was conducted on GDM patients admitted to a Grade III-A general hospital using convenience sampling between March and November 2021. The inclusion criteria were: (1) diagnosis of GDM according to the diagnostic criteria established by the International Association of Diabetes and Pregnancy Study Groups^[16]^; and (2) willingness to participate in the study. The exclusion criteria were: (1) presence of comorbidities such as hypertension, endocrine disorders, and cardiovascular diseases; (2) diagnosis of psychotic disorders such as schizophrenia, paranoid psychosis, or manic disorders; (3) presence of other pregnancy complications such as placenta previa or intrahepatic cholestasis of pregnancy; and (4) receiving insulin therapy. This study was approved by the ethics committee of the First Affiliated Hospital of Chongqing Medical University, Chongqing, China.

### 2.2. Procedures

Basic Characteristics Questionnaire: The researchers designed a questionnaire specifically for this study, comprising the following sections: (1) demographic characteristics, such as age, occupation, education, mode of delivery payment, and average annual income of the couple; (2) obstetric characteristics, including gravidity, parity, gestational age, gestational weight gain, oral glucose tolerance test results, and expected mode of delivery; and (3) other relevant information including family history of diabetes.

#### 2.2.1. Pregnancy Anxiety Scale (PAS)

This scale was used to assess anxiety levels in 2 groups of pregnant women. It was developed by Jiang Minhui in 2019^[16]^ and underwent rigorous evaluation, including item analysis, exploratory factor analysis, confirmatory factor analysis, and reliability and validity evaluation. The scale consists of 4 dimensions: delivery anxiety, self-anxiety, fetal anxiety, and general anxiety, with a total of 27 items. Respondents rated each item on a 5-point scale, reflecting the frequency of symptom occurrence: “1” = never, “2” = occasionally, “3” = sometimes, “4” = frequently, “5” = every day. The total score was the sum of the 27 items, with higher scores indicating greater pregnancy anxiety. The scale demonstrated excellent internal consistency with a Cronbach alpha coefficient of 0.95. PAS scores were recoded into binary values (0 or 1): original scores of 1 or 2 were recorded as 0, and scores of 3, 4, or 5 were recorded as 1.

#### 2.2.2. Marital Satisfaction Scale

The Marital Satisfaction Scale^[17]^ was specifically designed to assess satisfaction in marital relationships. It comprises 3 dimensions: marital emotions, marital cognition, and marital communication, with a total of 10 items. Respondents used a 4-point rating scale to indicate their level of agreement: “1” = completely disagree, “2” = disagree, “3” = agree, “4” = completely agree. A higher score indicates a higher level of satisfaction with marital relationships. The scale demonstrated strong internal consistency, as evidenced by Cronbach alpha coefficient of 0.93.

#### 2.2.3. GDM Knowledge Questionnaire

Sun Zhaona developed this questionnaire in 2017^[18]^ to evaluate knowledge related to GDM, covering disease mechanisms, outcomes, treatment methods, dietary management, exercise programmes, and self-care. The questionnaire comprises 6 dimensions, with 8 items in total. It employs a 5-point rating scale to assess participants’ understanding of the disease, using the following criteria: “1” = completely unaware, “2” = slightly aware, “3” = moderately aware, “4” = mostly aware, “5” = completely aware. The total score is the sum of the 8 items, with higher scores indicating a greater level of disease understanding. The questionnaire demonstrated good internal consistency with a Cronbach alpha coefficient of 0.84.

The content of the questionnaires was designed in an electronic form using the “Sojump” website (https://www.wjx.cn/). Participants were provided with QR codes for direct scanning, facilitating completion through WeChat. Upon submission, questionnaires were automatically transmitted to the Wenjuanxing platform. The questionnaires were distributed by the researchers themselves, using a standardized procedure to obtain informed consent. A total of 217 questionnaires were collected, but 2 questionnaires with a completion time of <100 seconds were excluded, resulting in 215 valid questionnaires and a response rate of 99%.

### 2.3. Statistical analysis

The sample size was determined using the 10EPV principle, considering multiple factors. Previous literature analyzing the factors influencing anxiety in GDM patients indicated approximately 15 to 20 factors.^[19–21]^ Therefore, the estimated sample size was 150 to 200 cases, and the final effective survey sample included 215 GDM patients.

An LCA model was established using Mplus 8.4 software. The PAS items were transformed into a binary scoring system (0, 1) by recording original scores of 1 and 2 as 0, and scores of 3, 4, and 5 as 1. Model fit assessment involved the following criteria: (1) log likelihood, Akaike information criterion, Bayesian information criterion, and adjusted Bayesian information criterion. Lower values indicate a superior model fit. (2) The Lo Mendell–Rubin adjusted likelihood ratio test and bootstrap likelihood ratio test were used to compare model fit among different numbers of latent classes. A significant *P*-value (*P* < .05) indicates that a model with k classes is a better fit than a model with k-1 classes. (3) Entropy was used to assess the accuracy of individual classification into respective classes, with values ranging from 0 to 1. Values closer to 1 indicate more precise classification. An entropy value of ≥ 0.80 indicates high classification accuracy.

Data analysis was performed using SPSS 23.0 statistical software (IBM, Armonk, NY). Normally distributed continuous variables are presented as mean ± standard deviation and were compared using analysis of variance for between-group comparisons. Non-normally distributed continuous variables are presented as medians (interquartile ranges) and were compared using the Kruskal–Wallis *H* test. Categorical variables were presented as counts and percentages. The chi-square test, analysis of variance, and Fisher exact test were used for univariate analysis of the latent classes. To explore the factors associated with anxiety latent classes in GDM patients, unordered multivariate logistic regression was used, including variables with a *P*-value < .05 from the univariate analysis in the multivariate logistic regression analysis. The significance level was set at *P* = .05.

## 3. Results

A total of 215 women with GDM were enrolled, with an average age of 31.33 ± 3.99 years. The patients’ average total PAS score was 59.18 ± 17.83, including dimensions of childbirth anxiety (18.51 ± 6.41), self-anxiety (14.16 ± 5.22), fetal anxiety (13.49 ± 4.63), and general anxiety (13.02 ± 4.01). The average SCR score was 28.83 ± 1.76, and the average GDM knowledge score was 29.03 ± 3.94 (Table 1).

**Table 1:** Basic characteristics (n = 215).

Variables	Classifications	Numbers or values
Age	<35 years	169
	≥35 years	46
Education	High school, associate degree and below	66
	Bachelor’s degree and above	149
Average annual household income	≤50,000	48
	50,000–100,000	42
	≥100,000	125
Payments methods	Maternity insurance	165
	Resident medical insurance	13
	Out-of-pocket	37
Parity	Primipara	131
	Multipara	84
Adverse obstetric history	Yes	6
	No	209
Planned mode of delivery	Planned vaginal delivery	94
	Planned cesarean section	77
	Undecided	44
Gestational age	≤32 Weeks	93
	32–36 + 6 Weeks	75
	≥37 Weeks	47
Blood glucose levels	1 elevated value	152
	2 or more elevated values	63
Family history of diabetes	Yes	88
	No	127
PAS score	Total PAS score	59.18 ± 17.83
	Childbirth anxiety dimensions	18.51 ± 6.41
	Self-anxiety dimensions	14.16 ± 5.22
	Fetal anxiety dimensions	13.49 ± 4.63
	General anxiety dimensions	13.02 ± 4.01
SCR score	-	28.83 ± 1.76
GDM knowledge score	-	29.03 ± 3.94

*Note*: In the variable “Blood glucose levels,” “1 elevated value” refers to having one of the following values elevated: fasting blood glucose, 1-hour postprandial blood glucose, or 2-hour postprandial blood glucose, exceeding the normal range. Unk >2 or more elevated values” refers to having 2 or all 3 of these values elevated above the normal range.

GDM = gestational diabetes mellitus, PAS = Pregnancy Anxiety Scale, SCR = Marital Satisfaction Scale.

**Figure 1.**
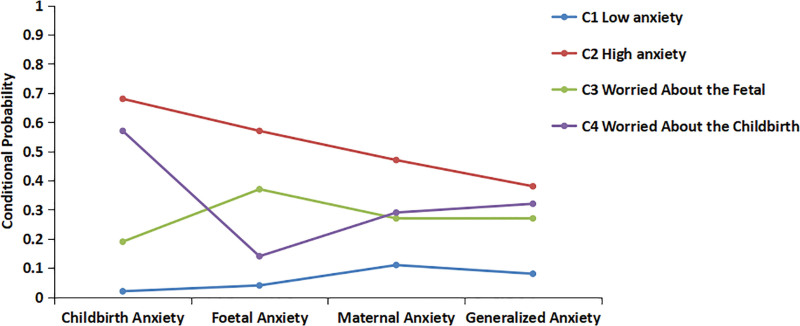
Distribution of 4 latent classes of anxiety in patients with GDM.

Four latent class models were used in this study. The model with 4 classes demonstrated the minimum Bayesian information criterion value and ideal entropy. Both the Lo Mendell–Rubin adjusted likelihood ratio test and bootstrap likelihood ratio test tests in the model with 4 classes produced statistically significant results (*P* < .05). Therefore, a 4-class model (C1, C2, C3, and C4) was selected for classification (Table 2). GDM patients were categorized into 4 potential categories: “C1: low anxiety” (n = 115, 54%), “C2: high anxiety” (n = 45, 21%), “C3: fetal concern” (n = 24, 11%), and “C4: delivery concern” (n = 31, 14%) (Fig. 1).

**Table 2:** LCA model fit for the symptom characteristics of anxiety (n = 215).

Model	K	Log likelihood	Akaike information criterion	Bayesian information criterion	Adjusted Bayesian information criterion	Entropy	Lo Mendell–Rubin adjusted likelihood ratio	Bootstrap likelihood ratio test	Class probabilities
1	8	−1916.415	3848.831	3875.796	3850.446	-	-	-	-
2	13	−1697.021	3420.042	3463.861	3422.666	0.931	438.789*	438.789*	0.69/0.31
3	18	−1644.87	3325.74	3386.412	3329.373	0.947	104.302†	104.302*	0.21/0.62/0.17
4	23	−1610.444	3266.888	3344.413	3271.53	0.94	68.852†	68.852*	0.54/0.21/0.11/0.14

*Note*: K represents the estimated number of parameters.

* *P* < .001.

† *P* < .05.

The 4 potential categories of anxiety in GDM patients exhibited significant differences in terms of educational background, parity, adverse pregnancy history, mode of delivery, blood glucose value change, family history of diabetes, relationship assessment scale, and disease awareness scores (*P* < .05) (Table 3). Multivariate logistic regression analysis showed that, compared with low anxiety, education (OR = 0.30, 95% CI: 0.13–0.70, *P* = .005), family history of diabetes (OR = 0.36, 95% CI: 0.17–0.77, *P* = .009), blood glucose value change (OR = 20.95, 95% CI: 8.97–48.90, *P* < .001), delivery mode schedule (OR = 22.35–22.40, 95% CI: 5.56–82.80, *P* < .001), GDM knowledge score (OR = 0.86, 95% CI: 0.78–0.95, *P* = .002), and marital relationship scale score (OR = 0.61, 95% CI: 0.49–0.76, *P* < .001) were independently associated with high anxiety. Patients’ number of births (OR = 3.16, 95% CI: 1.49–6.70, *P* = .003), education (OR = 0.42, 95% CI: 0.18–0.95, *P* = .037), blood glucose value change (OR = 4.49, 95% CI: 1.77–11.37, *P* = .002), delivery mode schedule (OR = 8.35–11.34, 95% CI: 2.79–34.63, *P* < .001), and marital relationship scale score (OR = 0.77, 95% CI: 0.62–0.95, *P* = .014) were independently associated with fetal concern. Education (OR = 0.39, 95% CI: 0.17–0.90, *P* = .027), family history of diabetes (OR = 0.27, 95% CI: 0.13–0.58 *P* < .001), blood glucose value change (OR = 6.28, 95% CI: 2.59–15.22, *P* < .001), delivery mode schedule (OR = 4.31–7.05, 95% CI: 1.56–19.73, *P* < .05), GDM knowledge score (OR = 0.84, 95% CI: 0.76–0.92, *P* < .001), and marital relationship scale score (OR = 0.57, 95% CI: 0.45–0.71, *P* < .001) were independently associated with delivery concern (Table 4 and Table S1, Supplemental Digital Content, http://links.lww.com/MD/N322).

**Table 3:** Comparison among the 4 latent classes of anxiety (n=215).

Project [example (%)]	Low anxiety (n = 115)	High anxiety (n = 45)	Worried about the fetal (n = 24)	Worried about the childbirth (n = 31)	Test statistic	*P*-value
Age					3.7*	0.3
<35 yr	87 (75.7%)	38 (84.4%)	17 (70.8%)	27 (87.1%)		
≥35 yr	28 (24.3%)	7 (15.6%)	7 (29.2%)	4 (12.9%)		
Education						
Secondary school, vocational degree, and below	27 (23.5%)	21 (46.7%)	7 (29.2%)	11 (35.5%)	8.57*	0.036
Undergraduate degree and above	88 (76.5%)	24 (53.3%)	17 (70.8%)	20 (64.5%)		
Average annual household income of the couple						
≤50 000	24 (20.9%)	12 (26.7%)	6 (25.0%)	6 (19.4%)	/†	0.504
50 000–100 000	25 (21.7%)	6 (13.3%)	2 (8.3%)	9 (29.0%)		
≥100 000	66 (57.4%)	27 (60.0%)	16 (66.7%)	16 (51.6%)		
Payment methods						
Maternity insurance	85 (51.5%)	35 (21.1%)	19 (11.5%)	26 (15.8%)	/†	0.84
Resident health insurance	9 (69.2%)	3 (23.1%)	1 (7.7%)	0		
Out-of-pocket	21 (56.8%)	7 (18.9%)	4 (10.8%)	5 (13.5%)		
Parity						
Primipara	63 (54.8%)	36 (80.0%)	9 (37.5%)	23 (74.2%)	16.53*	<0.01
Multipara	52 (45.2%)	9 (20.0%)	15 (62.5%)	8 (25.8%)		
Choice of delivery method						
Expected vaginal delivery	77 (67.0%)	4 (8.9%)	5 (20.8%)	8 (25.8%)	/†	<0.01
Expected cesarean section	25 (21.7%)	28 (62.2%)	12 (50.0%)	12 (38.7%)		
Undecided	13 (11.3%)	13 (28.9%)	7 (29.2%)	11 (35.5%)		
Gestational weeks						
≤32 wk	47 (40.9%)	20 (44.4%)	11 (45.8%)	15 (48.4%)	3.61*	0.73
32–36 + 6 wk	40 (34.8%)	14 (31.1%)	8 (33.3%)	13 (41.9%)		
≥37 wk	28 (24.3%)	11 (24.4%)	5 (20.8%)	3 (9.7%)		
Adverse pregnancy history						
Yes	0	5 (11.1%)	0	1 (3.2%)	/†	<0.01
No	115 (100%)	40 (88.9%)	24 (100%)	30 (96.8%)		
Changes in blood glucose levels						
Elevated value in 1 category	103 (89.6%)	14 (31.1%)	17 (70.8%)	18 (58.1%)	56.19*	<0.01
Elevated values in 2 or more categories	12 (10.4%)	31 (68.9%)	7 (29.2%)	13 (41.9%)		
Family history of diabetes						
Yes	34 (29.6%)	27 (60.0%)	11 (45.8%)	16 (51.6%)	14.6*	<0.01
No	81 (70.4%)	18 (40.0%)	13 (54.2%)	15 (48.4%)		
Relationship Assessment Scale Score	31 (31, 31)	27 (27, 27)	32 (32, 32)	21 (21, 21)	178.48‡	<0.01
Disease Awareness Score	30 (30, 37)	27 (27, 27)	227 (27, 27)	26 (26, 26)	154.78‡	<0.01

* Chi-square value.

† Fisher’s exact probability test did not show statistical significance.

‡ *H*-value.

**Table 4:** Multivariate logistic regression for 4 latent classes of anxiety.

Variables	OR value	95% CI	*P*-value
*High anxiety versus low anxiety*			
Education	0.30	0.13–0.70	.005
Family history of diabetes	0.36	0.17–0.77	.009
Changes in blood glucose levels	20.95	8.97–48.90	<.001
Choice of delivery method (expected cesarean section)	22.40	6.06–82.80	<.001
Choice of delivery method (Undecided)	21.35	5.56–81.99	<.001
Disease awareness score	0.86	0.78–0.95	.002
Relationship assessment scale score	0.61	0.49–0.76	<.001
*Worried about the fetal versus low anxiety*			
Parity	3.16	1.49–6.70	.003
Education	0.42	0.18–0.95	.037
Changes in blood glucose levels	4.49	1.77–11.37	.002
Choice of delivery method (expected cesarean section)	8.35	2.79–24.97	.001
Choice of delivery method (undecided)	11.34	3.71–34.63	.001
Relationship assessment scale score	0.77	0.62–0.95	.014
*Worried about the childbirth versus low anxiety*			
Education	0.39	0.17–0.90	.027
Family history of diabetes	0.27	0.13–0.58	.001
Changes in blood glucose levels	6.28	2.59–15.22	<.001
Choice of delivery method (expected cesarean section)	4.31	1.56–11.93	.005
Choice of delivery method (undecided)	7.05	2.52–19.73	<.001
Disease awareness score	0.84	0.76–0.92	<.001
Relationship assessment scale score	0.57	0.45–0.71	<.001

*Note*: The table only displays variables that have statistical significance.

## 4. Discussion

Anxiety during pregnancy can lead to postpartum depression, fetal dysplasia, premature delivery, increased risk of neonatal diseases, and even suicidal ideation.^[6,7]^ Therefore, research on anxiety-related factors in GDM patients is essential. Sonaglioni et al^[22]^ reported that thoracic structural importance impacts cardiac parameters and symptoms during pregnancy, suggesting that patients with GDM who have a narrower chest diameter are more likely to suffer from anxiety.

LCA categorized the anxiety of GDM patients into 4 potential categories: low anxiety (54%), high anxiety (21%), fetal concern (11%), and delivery concern (14%). Education, family history of diabetes, blood glucose change, delivery mode schedule, GDM knowledge score, and marital relationship scale score were found to be associated with anxiety. This study highlights the significant differences in anxiety characteristics among GDM patients across various categories.

The occurrence rate of the “Worried About Fetal” category was 11%, with anxiety in this group stemming from concerns about the disease’s impact on the fetus.^[23]^ A study conducted by De Asis-Cruz et al demonstrated a correlation between maternal prenatal anxiety and impaired fetal brain functional connectivity, leading to early functional deviations, developmental abnormalities, congenital defects, and malformations. Thus, in clinical practice, it is advisable to guide patients to undergo regular antenatal checkups and educate them about methods for monitoring fetal safety at home. While current evidence does not suggest that vertical transmission is a noteworthy risk, patient concerns have not diminished over time in the absence of population-based evidence. To ensure intrauterine safety and alleviate the concerns of pregnant women, remote fetal heart monitoring has been suggested.^[24]^ A study showed that strengthening patients’ self-management abilities, correcting disease perception, and reducing anxiety levels can help mitigate the impact on the fetus.

The occurrence rate of the “Worried About Childbirth” category was 14%. This study revealed that the “High Anxiety,” “Worried About Fetal,” and “Worried About Childbirth” groups exhibited a lower probability of opting for vaginal delivery compared to elective cesarean section. Compared to natural childbirth, cesarean section is considered a safer option for both the mother and the infant. It has been reported that fear of pain and anxiety can stimulate the release of substances such as norepinephrine, adrenaline, and dopamine, leading to increased excitability of the sympathetic nervous system and reduced psychological adaptability to natural childbirth. Consequently, a preference for cesarean section arises.^[25]^ In China, the relaxation of the national birth policy has led to an increase in the rate of first-time cesarean sections, subsequently contributing to higher cesarean section rates for second and third pregnancies. Despite efforts by the country to control the cesarean section rate, the results have been unsatisfactory. In some regions of China, the cesarean section rate exceeds 50%, as indicated by incomplete statistics.^[26]^ Therefore, it is crucial to decrease the rate of first-time cesarean sections to lower the overall cesarean section rate. Healthcare professionals should strengthen the management of primiparous women, ensure that fetal weight is within the appropriate range, and prevent macrosomia. Additionally, promoting the benefits of natural childbirth and offering related courses during the mid to late stages of pregnancy, such as prenatal yoga classes, birthing dance, and online exercise camps, can be beneficial.

The prevalence of the “High Anxiety” category was found to be 21%. In this study, all anxiety categories displayed remarkably elevated levels of anxiety, suggesting that patients are concerned not only about the fetus and the process of childbirth but also about their own health. According to previous reports,^[27]^ GDM patients are prone to developing cardiovascular diseases, lipid abnormalities, and postpartum metabolic disorders. Currently, there is no standardized model for the care of GDM patients; however, precise and tailored care can be provided by considering aspects such as the patient’s physiology, psychology, diet, and exercise.^[28]^ Encouraging patients to proactively cope with their situation can effectively mitigate anxiety levels and minimize subsequent impacts on both the mother and the fetus.

The results of this study revealed that the “High Anxiety,” “Worried About Fetal,” and “Worried About Childbirth” categories exhibited an increased likelihood of experiencing dramatically abnormal blood glucose changes. This suggests that patients with significantly abnormal blood glucose levels might express notable concerns about childbirth, as well as their own health and the well-being of their fetus. Moreover, anxiety itself serves as a risk factor for GDM because it stimulates the sympathetic adrenal medullary system; promotes ACTH secretion; increases levels of glucocorticoids, insulin, and catecholamines; and ultimately accelerates gluconeogenesis and glycogenolysis, further increasing blood glucose levels.^[29]^ This indicates an interaction between changes in blood glucose levels and anxiety. Thus, the alleviation of anxiety may help reduce the incidence of GDM. A study revealed that non-pharmacological management, including moderate aerobic exercise and interpersonal communication, plays a key role in the control of blood glucose levels in GDM patients.^[30]^ These measures could assist patients in maintaining blood glucose levels within an appropriate range, thereby reducing the incidence of adverse maternal and neonatal outcomes while ensuring the safety of both the mother and child.

The study also revealed that the “High Anxiety” and “Worried About Childbirth” categories exhibited an increased likelihood of having a family history of diabetes. A previous study also showed that a family history of diabetes is an independent risk factor for GDM.^[31]^ Early assessment of GDM risk during pregnancy, along with health education and early interventions for high-risk individuals, plays a pivotal role in mitigating the incidence of GDM in China.^[32]^ Therefore, in clinical practice, patients with a family history of diabetes should undergo continuous monitoring of blood glucose levels from early pregnancy, receive nutritional and exercise guidance, and adhere to strict weight control.

The results of this survey indicated that the “High Anxiety,” “Worried About Fetal,” and “Worried About Childbirth” categories had a higher probability of having a high school education or below. This finding is consistent with previous studies that showed education significantly influences participants’ anxiety. Individuals with lower education levels may face limited access to disease-related knowledge, leading to inadequate understanding of the disease, which, in turn, may result in excessive worries about personal and fetal safety, leading to heightened anxiety levels. Furthermore, this study revealed that the “Worried About Fetal” category had a higher probability of being multiparous. Compared to primiparous women, multiparous women face an elevated risk of adverse maternal and neonatal outcomes during the perinatal period.^[33]^ Hu et al^[34]^ suggested that parity and gravidity are risk factors for anxiety during pregnancy. However, Li et al^[35]^ reported that first-time pregnant patients may exhibit insufficient knowledge, lack mental and physical preparedness, and experience excessive worries about childbirth and newborn care, leading to increased psychological stress and anxiety. These discrepancies may be attributed to variations in the proportions of different parity statuses within the sample and regional disparities. Therefore, further research with a larger sample size is required. In the clinical care of GDM patients, it is crucial to prioritize the management of high-risk women and employ various educational methods and approaches to meet the needs of patients from different cultural backgrounds.

The results of this study showed that the “High Anxiety,” “Worried About Fetal,” and “Worried About Childbirth” categories had lower scores on the Couples Relationship Scale. These findings are consistent with those of a previous study, which indicated that a harmonious marital relationship serves as an important protective factor for pregnant women, alleviating pregnancy-related stress and fostering confidence in childbirth.^[25]^ When facing adverse emotions such as anxiety, pregnant women need family support, with the husband as the core of this support, exerting a direct impact on the physical and mental health of the expectant mother.^[36]^ Moreover, a positive marital relationship is associated with enhanced emotional bonding, promoting the release of endogenous oxytocin,^[37]^ which directly causes uterine contractions,^[38]^ thereby facilitating the onset of labor. A study conducted by Li et al^[39]^ demonstrated that a positive marital relationship acts as a protective factor against labor pain, which is a significant factor hindering childbirth. Parturients with a positive marital relationship tend to receive more support and care from their husbands, which raises the pain threshold and reduces the perception of labor pain. Additionally, the COVID-19 pandemic has resulted in restrictions on companionship during delivery, impacting patients’ birth plans and elevating the risk of anxiety during pregnancy.^[40]^ To cope with this adverse situation, husbands should play a more active role in managing pregnant patients. They should acquire a comprehensive understanding of disease management methods and behaviors, rectify misconceptions, and contribute to blood glucose control.^[41]^ Therefore, a positive marital relationship plays a crucial role in alleviating anxiety related to fetal concerns, childbirth, and self-concern. In the management of GDM patients during pregnancy, interventions that prioritize couples, such as online prenatal classes and music therapy,^[42]^ along with dyadic coping interventions,^[41,43]^ can be utilized to reduce patient anxiety and improve disease outcomes and prognosis.

The results of this study indicate that patients in the “High Anxiety” and “Worried About Childbirth” categories exhibited reduced levels of disease awareness. Diminished self-management abilities are associated with reduced disease knowledge, hindering patients’ capacity to control their diet and engage in physical activity,^[44]^ leading to suboptimal blood glucose control. Anxiety is a manifestation of psychological stress, and when individuals perceive events as uncontrollable and unpredictable, their fear and anxiety intensify, resulting in irrational anxiety.^[45]^ Therefore, in the management of GDM patients, it is important to equip them with knowledge about the disease pathogenesis, prognosis, treatment methods, self-care, dietary guidelines, and exercise programmes.

In this study, a comprehensive investigation of GDM patients was conducted, exploring the potential risk factors and protective factors for GDM-related anxiety. Additionally, tailored strategies were provided for different categories of patients with GDM, thus offering a theoretical basis for the management of these patients. However, this study had several limitations. Due to the cross-sectional nature of the study, causal relationships could not be determined. Furthermore, this was a single-center, single-race study, which may introduce sample bias. The sample size was also relatively small. Therefore, future multicentre prospective studies with larger sample sizes are warranted. Finally, we did not assess the chest morphology of the GDM women; future follow-up studies could consider including this aspect to explore its relationship with anxiety.

In conclusion, LCA categorized GDM patients with anxiety into 4 potential categories: low anxiety (54%), high anxiety (21%), worried about the fetus (11%), and worried about delivery (14%). Education, family history of diabetes, changes in blood glucose, delivery mode schedule, GDM knowledge score, and marital relationship scale score may be associated with anxiety. Considering the distinct characteristics of patients with GDM, tailored intervention measures provided in this study should be implemented in clinical care management.

## Acknowledgments

The authors would like to express their gratitude to all pregnant women for their cooperation in the survey.

## Author contributions

**Project administration:** Fulan Wang.

**Writing – original draft:** Hong Qin.

**Writing – review & editing:** Ling Wang.

## Supplementary Material
