# Supplementary material for: Categories of anxiety based on latent class analysis in gestational diabetes mellitus and associated factors: A questionnaire-based, cross-sectional study
Source: Medicine (Baltimore). 2024 Aug 9;103(32):e39168. doi: 10.1097/MD.0000000000039168 (PMC11315481; doi:10.1097/MD.0000000000039168)
Supplement: Supplementary file 1 [file medi-103-e39168-s001.docx]

**Table S1 Assignment of independent variables for multivariate logistic regression**

| Variables | Assignment Conditions |
| --- | --- |
| Education | Secondary School and Below = 1, Bachelor's Degree and Above = 2 |
| Parity | Primipara = 1, Multipara = 2 |
| Adverse Pregnancy History | Yes = 1, No = 2 |
| Choice of Delivery Method | Taking expected vaginal delivery as the reference, dummy variables were set for expected cesarean section (FS1=1, FS2=0) and "to be determined at the time" (FS1=0, FS2=1). |
| Changes in Blood Glucose Levels | Elevated value in one category = 1, elevated values in two or more categories = 2 |
| Family History of Diabetes | Yes = 1, No = 2 |
| Relationship Assessment Scale Score | Original values have been used. |
| Disease Awareness Score | Original values have been incorporated. |
